# LinkedIn to Get In: embedding learning activities to support student career development through online professional networking

**DOI:** 10.1099/acmi.0.001125.v3

**Published:** 2026-01-14

**Authors:** Mahmood M. Alam, Fiona K. Stubbs, Anna E. Nousek-McGregor, Leighann Sherry

**Affiliations:** 1 School of Infection & Immunity, College of Medical, Veterinary and Life Sciences, University of Glasgow, Glasgow, UK; 2 Careers, Employability & Opportunity, University of Glasgow, Glasgow, UK; 3 School of Biodiversity, One Health & Veterinary Medicine, College of Medical, Veterinary and Life Sciences, University of Glasgow, Glasgow, UK

**Keywords:** employability, career awareness, curriculum, networking, LinkedIn, undergraduate

## Abstract

The extent to which individuals interact online has expanded in recent years, with online networking playing a major aspect of most people’s lives. With employers relying on online searches when evaluating job candidates, the development of a positive professional online presence has become an important aspect in most sectors and potentially a challenge for students preparing to enter the workplace. LinkedIn is a globally recognized networking site, enabling individuals to interact within a professional environment. However, it remains uncertain whether students are aware of its benefits and are confident in using it. Alongside other Life Science degree programmes at the University of Glasgow, staff within the Microbiology and Immunology programmes initiated closed LinkedIn groups, which were limited to staff and current or former students of the relevant degree. The aim of these groups was to promote student awareness of the diversity of career roles available post-university and enable students to network in their chosen field, as levels of student engagement with this platform and how it can be utilized by students for professional networking remained unclear. As part of our ‘Linked to Get In’ workshop, students connected and conducted an interview with an alumnus of their degree, allowing them to develop their networking skills with professionals in their field and enabling exploration of career prospects, prior to presenting their findings to their peers. Confidence in using LinkedIn for networking increased following this session, with all students agreeing that the inclusion of such a workshop in the curriculum is useful for exploring employability options. Moreover, students had increased awareness of the wider benefits the platform had to offer, and it was not only useful for job searching. Our findings show that LinkedIn has the potential for being an effective platform to enable undergraduate students to engage with professionals in their discipline, with the closed format providing a trusted space for students to enhance their networking and communication skills, whilst exploring the career diversity open to them.

## Data Summary

The authors confirm that all questionnaire data have been provided through a supplementary data file.

## Introduction

The extent to which individuals interact in an online environment has expanded considerably over the last 20 years, with up to five billion people globally now using social media [1]. Given the potential global reach and immediacy of the online environment, the role of online platforms for career advancement can be considerable, providing a highly beneficial and practical way of connecting professionals regardless of location and sharing news, opportunities and updates to an extremely large audience worldwide. LinkedIn is the largest online professional networking platform and can be utilized for career exploration and advancement, recruitment, skill development and mentorship opportunities, particularly outside academia [2,4]. In recent years, in response to the evolving employment landscape, an increasing number of higher education institutions have embedded employability into their educational strategies [5], aiming to equip students with academic knowledge, as well as the skills and experiences required to succeed in future careers. However, initiatives like this can only be helpful if students’ understanding of the employment routes open to them is clear. It is common for students to feel uncertain of their career options whilst studying [6], meaning they cannot fully engage with and utilize the employability teaching embedded into their curriculum. Therefore, it is imperative that educators utilize all opportunities available to them to equip students with the knowledge and opportunities to facilitate their career ambitions.

Social networks have great potential for crossing barriers to education, helping to create a level playing field for all and increasing social capital [7,8]. Given the extensive use of social networking platforms and the need to embed employability into the curriculum, exploring how professional platforms can be utilized to improve students’ networking skills is important, in turn supporting the advancement of their careers. The use of LinkedIn provides undergraduate students with a platform which they can use to connect with professionals in areas from future careers [9,10]. Some institutions are already developing ways to scaffold use of LinkedIn into the curricula [11,12]; however, despite the creation of a professional online presence becoming an important aspect of careers across most industries, whether undergraduate students feel confident and equipped to use LinkedIn as a method of exploring career opportunities and networking with established professionals in their field of interest remains unclear.

The aim of this study was to explore student perspectives of using LinkedIn as a networking platform, investigating their understanding of how it can be used to explore career pathways open to them. It also aimed to evaluate whether a structured initial engagement with the platform can increase student confidence in using the platform, ultimately directed at promoting its use post-university. Finally, it aimed to increase student awareness of the different potential roles available post-university by arranging small-group interviews between current students and recent former graduates of the degree. By examining student reflections on these activities, we hope to further enhance ways of embedding employability into the curriculum that highlight the use of digital tools to support post-university career development.

## Methods

### Data collection

Undergraduate students in the penultimate year of their degree within the School of Infection and Immunity at the University of Glasgow were the participants in this project, as this is the stage when Life Sciences students specialize in their chosen degree programme. Students enrolled on the Year 3 BSc (Hons) Microbiology (*n*=64) or Immunology (*n*=80) courses over the academic years 2022–2023 and 2023–2024 were asked to complete anonymous online questionnaires before and after they attended a ‘LinkedIn to Get In’ workshop, with a response rate in the pre- (*n*=46) and post- (*n*=25) questionnaires of 31.9 and 17.4%, respectively. These questionnaires consisted of a combination of Likert scale and open-ended questions. Quantitative analysis was performed on Likert scale questions to understand student awareness and usage of the professional networking platform LinkedIn, including their confidence in using it for networking and their familiarity with the platform features to aid career advancement. In addition, thematic analysis was conducted on open-ended responses to ascertain if students understood the benefits offered by LinkedIn.

### Activity design

**Fig. 1.**
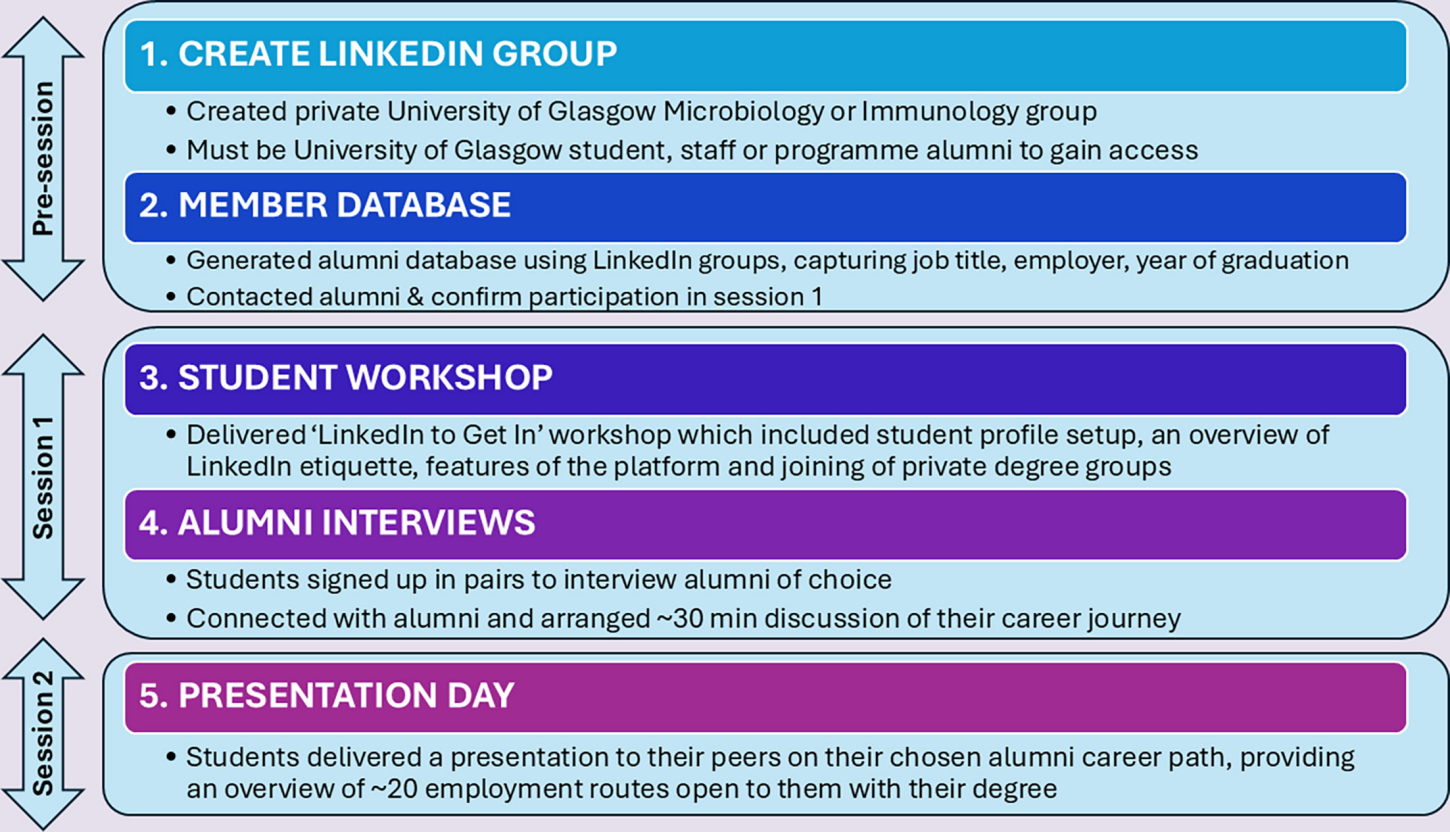
Summary of the key stages of the ‘LinkedIn to Get In’ employability workshop. The pre-session was staff-led and included the creation of LinkedIn groups (Step 1) and the generation of an alumni member database (Step 2). Session 1 supported the setup of undergraduate profiles (Step 3), followed by students conducting an interview with a chosen alum (Step 4). Session 2 was a presentation day, where students presented the career path of their chosen alum to their peers (Step 5).

The ‘LinkedIn to Get In’ workshop was conducted across two teaching sessions, with an overview of the design and delivery shown in Fig. 1.

Prior to the first session, private LinkedIn groups for students from the Microbiology and Immunology programmes were set up, restricted to current and former students of the associated courses and staff teaching on them. The purpose of using a closed group format was to provide a trusted space for students to network and build connections with those who have undertaken the same undergraduate programme at the University of Glasgow. Group membership was collated into a database detailing alumni job profiles, showcasing a range of Microbiology and Immunology specific roles as well as non-science positions our alumni enter. For these cohorts, the career routes explored included teaching, banking analyst, software engineer, NHS practitioners, quality assurance, research and development scientists and technicians working in oil and gas, university and pharmaceutical sectors.

This database was then used by staff to contact graduates to confirm their willingness to be interviewed by current students.

The first timetabled session of this exercise (session 1) was run as a workshop (Fig. 1), introducing students to the professional networking platform, including an overview of its functionality, guidance on how to create individual profiles and a discussion of online networking etiquette, particularly aimed at clarifying the difference between professional networking in comparison to social use of online platforms.

During session 1, students used their new account to join a closed University of Glasgow Microbiology or Immunology LinkedIn group and, in pairs, identified an alum whose role or organization resonated with their personal aspirations from the pre-populated member database and reviewed their career journey available on LinkedIn. Students connected with their chosen alum via this platform during session 1 and were supported in writing their connection requests. Moreover, this workshop also explored how students can contribute to their closed groups and wider LinkedIn community, providing examples of content that is appropriate for undergraduates to share with their network and how to interact with professionals in their field. Following the workshop, students were tasked with continuing to communicate with alumni and arranging to interview them for ~30 min about their career pathway, current role and employer’s profile. This provided an opportunity to network with professionals in their field, understanding the requirements and challenges faced when navigating their career post-graduation and receiving guidance on what students should be doing at university to help build the skills sought by employers.

Although students were asked to create a LinkedIn account to enable them to participate in this networking session, it was not mandatory. Despite no students stating they did not wish to create an account, measures were put in place to overcome this scenario if it were to arise. For example, students would be supported to connect with their chosen alumni via email, allowing them to arrange their interview via another platform and ensuring they are not disadvantaged in comparison to their peers.

Approximately 6 weeks after session 1, working in pairs, students prepared a short oral presentation on their chosen alum and presented their findings in person to their peers during session 2. Each pair presented their reflections on the alumnus’ career path, including the purpose and culture of the employing organization, the alum’s role and responsibilities within that organization and the skills required to reach that current position. By engaging through alumnus’ experience, this approach introduced current students to ~20 diverse career paths, covering potential directions within and outside of science and within and outside of academia, demonstrating the range of opportunities available with their degree and offering insights into the recruitment process within those organizations.

Following the ‘LinkedIn to Get In’ workshop, students (*n*=25) completed a post-workshop questionnaire to assess changes in their confidence in using LinkedIn, including their contribution to closed groups and to evaluate the workshop’s effectiveness in enhancing their networking ability.

## Results

### Effects of the workshop

**Fig. 2.**
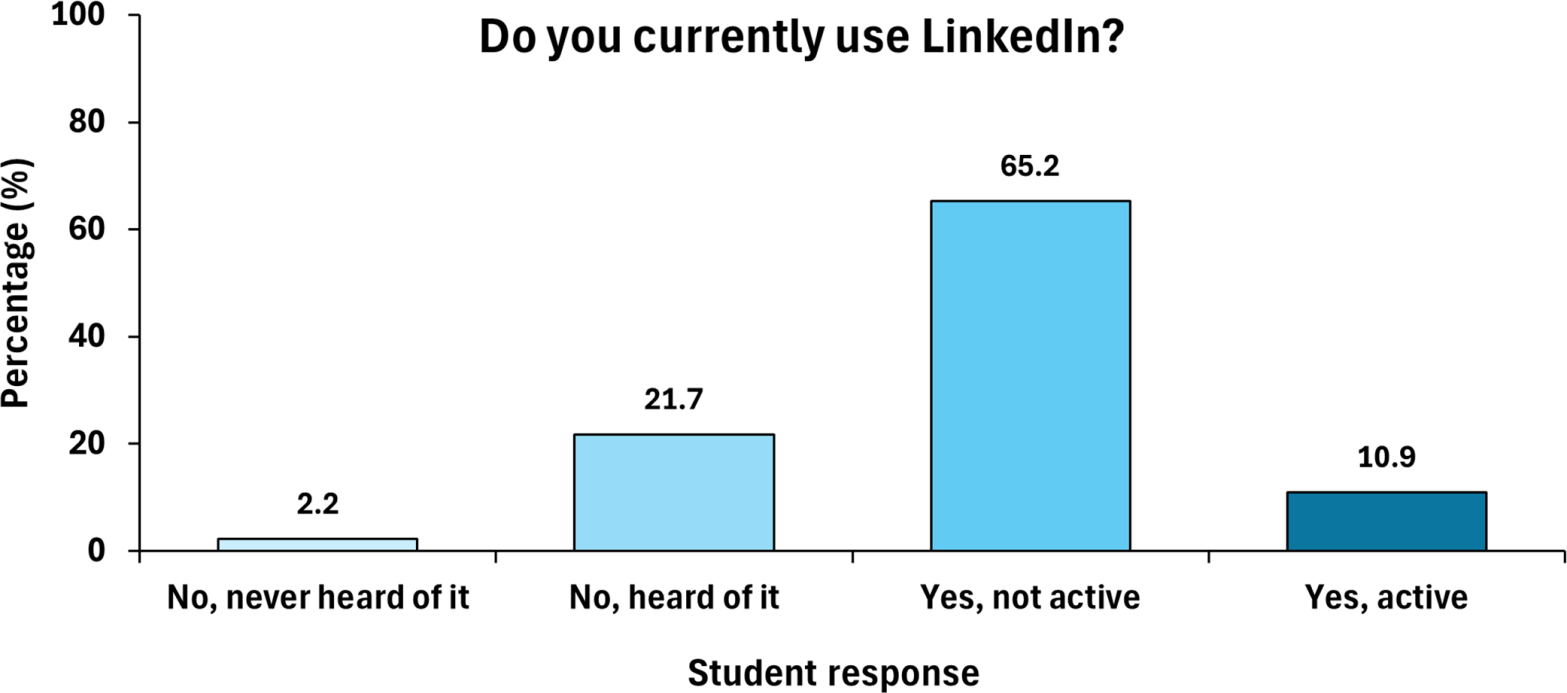
Pre-questionnaire responses (*n*=46) from Year 3 Microbiology and Immunology students on their current usage of LinkedIn. These responses show some awareness but very little current usage.

Very few students (10.9%) had actively engaged with LinkedIn prior to the Year 3 workshop, although 65% of the student participants had a LinkedIn profile prior to it, demonstrating good levels of awareness of the existence of the platform (Fig. 2).

**Fig. 3.**
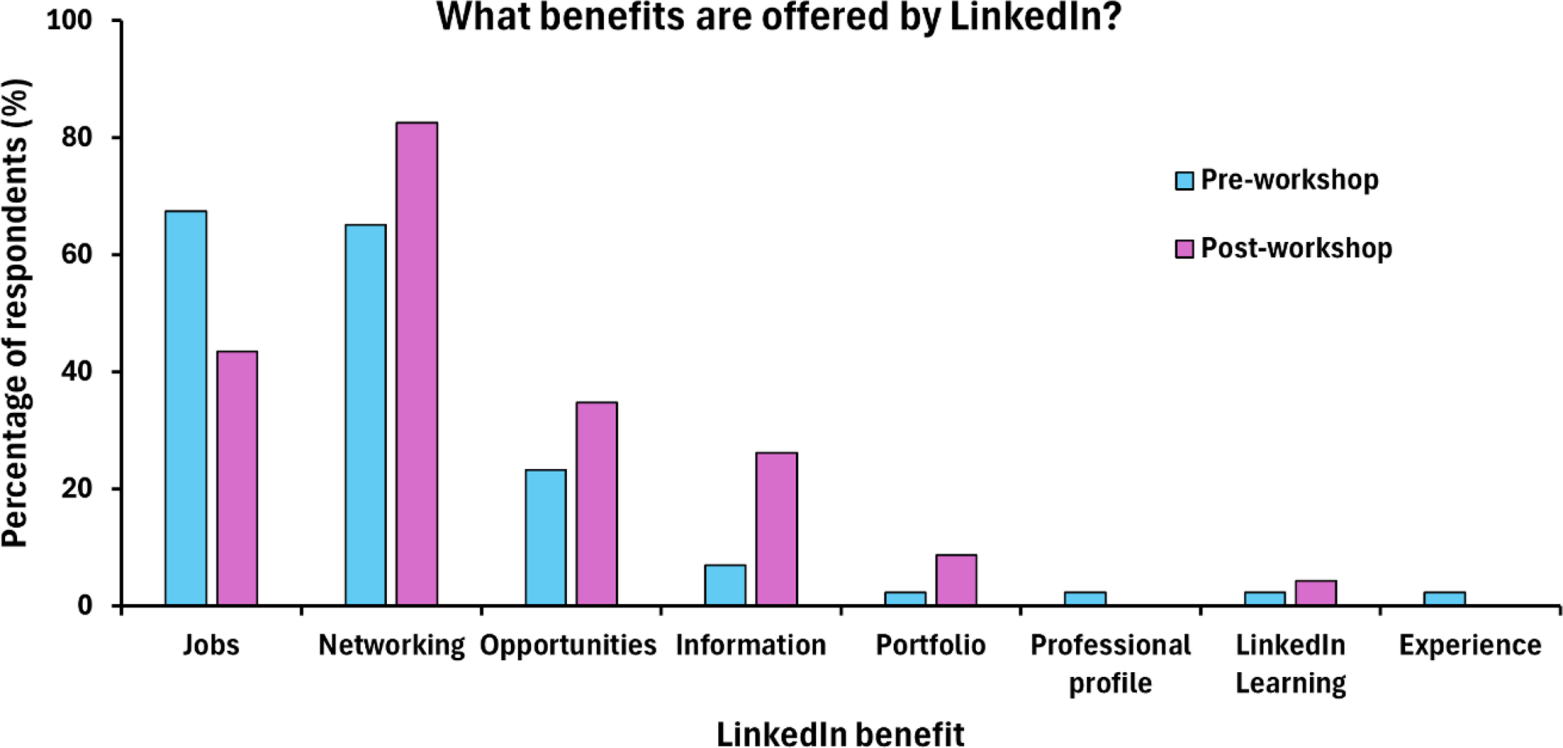
Students’ responses on understanding the benefits of using LinkedIn. The bar chart shows the percentage of respondents to the survey question on ‘What benefits are offered by LinkedIn?’, with different benefits stated by students. Light blue bars represent responses before the ‘LinkedIn to Get In’ workshop (*n*=43) and pink bars represent responses following the completion of the workshop (*n*=23).

Before the workshop, students mostly viewed LinkedIn as a job-posting or networking platform, with 67.4 and 65.1% of students listing these as benefits, respectively (Fig. 3). Other benefits that students were aware of before the workshop were ‘Opportunities’ (23.3%) and ‘Information’ (7%). A few participants were also aware of the learning resources available through the platform and how they could be utilized as an online portfolio tool.

Following the workshop, responses on the benefits offered by LinkedIn changed, with a lower percentage of students citing ‘Jobs’ (43.5%) as the main driver for using the platform. However, a higher proportion of students recognized the wider advantages of LinkedIn, with ‘Networking’ increasing by 17.5%, ‘Opportunities’ by 11.5% and ‘Information’ by 19.1%. The percentage of students now acknowledging the importance of having an online portfolio was up 6.4% from the pre-questionnaire. Moreover, open-ended responses provided insight into the benefits that students now associated with having a LinkedIn account.

*‘I can now see what other people who studied my course are doing professionally’.*

*‘Recruiters seeing your skills and contacting you about jobs’.*

*‘Opportunity to reach out to professionals in an interested subject area and potential for job opportunities and placements at larger or more private companies that do not advertise’.*

**Fig. 4.**
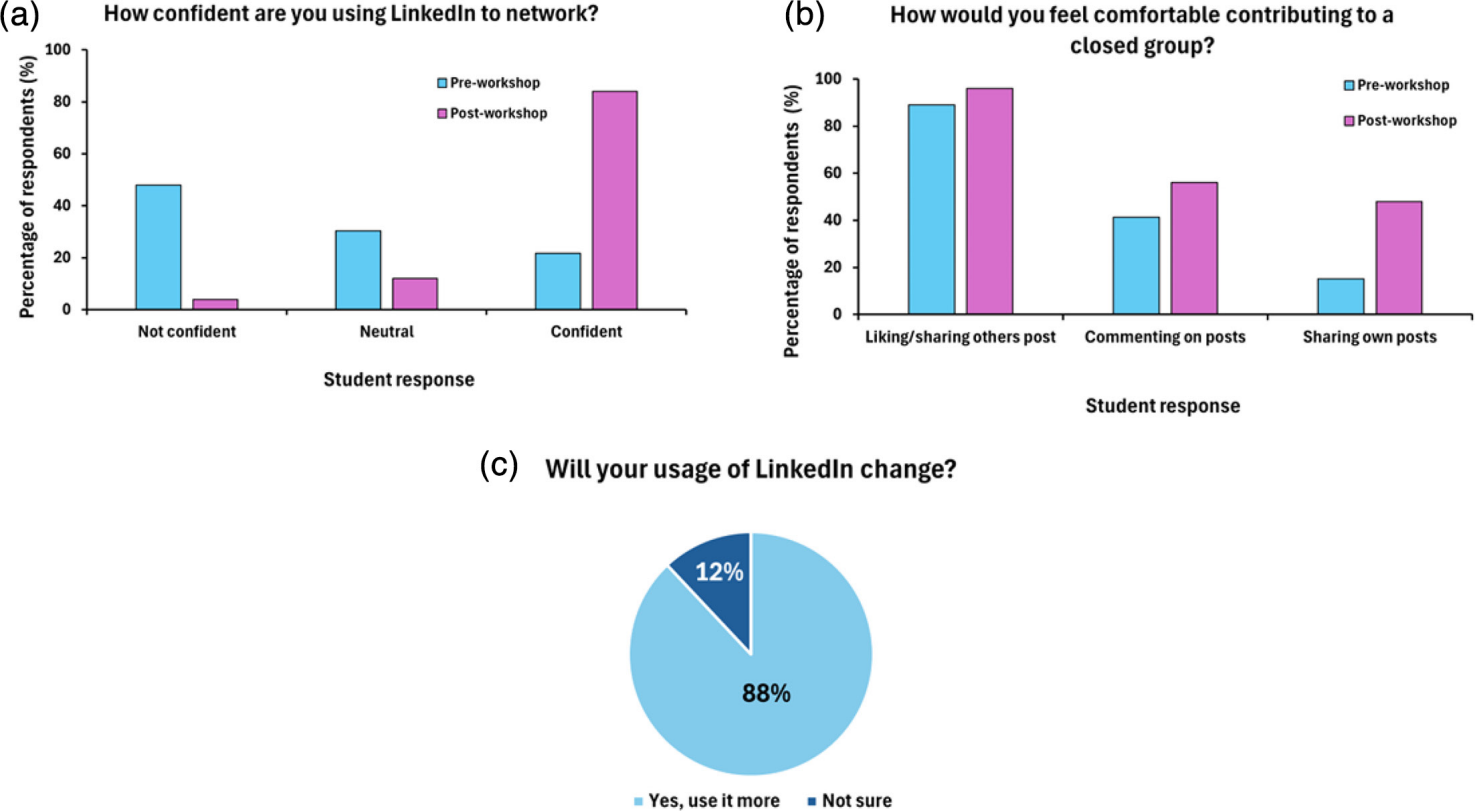
Students’ responses to their confidence in using the LinkedIn platform. Percentage of respondents to the questions (a) ‘How confident are you in using LinkedIn?’ and (b) ‘How would you feel comfortable contributing to a closed group?’. Light blue bars represent responses before the ‘LinkedIn to Get In’ workshop [*n*=46 for (a) and *n*=44 for (b)] and pink bars represent responses after the workshop (*n*=25). Percentage of students considering the question (**c**) ‘Will your use of LinkedIn change?’, with the area in light blue representing the percentage of students selecting ‘Yes’ and area in dark blue the students selecting ‘not sure’.

Prior to structured training, student confidence in using the platform was generally low, with 47.9% of students reporting ‘not confident’ in the survey and only 21.7% expressing confidence (Fig. 4a).

Confidence in using LinkedIn to network increased to 84% following the workshop, with the number of students lacking confidence decreasing to 4%. Similarly, students unsure about their confidence in using LinkedIn decreased from 30.4 to 12%, which overall highlights the success and impact of embedding networking opportunities into the curriculum.

Despite an increase in confidence, the level of comfort students felt in using a University of Glasgow degree specific group for networking still remained unclear. The percentage of students saying they would ‘share or like others' posts’ remained similar pre- (93.2%) and post-workshop (96%), as shown in Fig. 4(b). However, the proportion of students indicating post-workshop they would ‘comment on others’ posts’ increased from 43.1 to 56%, and importantly, students were more willing to write their own posts on completion of the workshop, increasing from 15.9 to 48%.

**Fig. 5.**
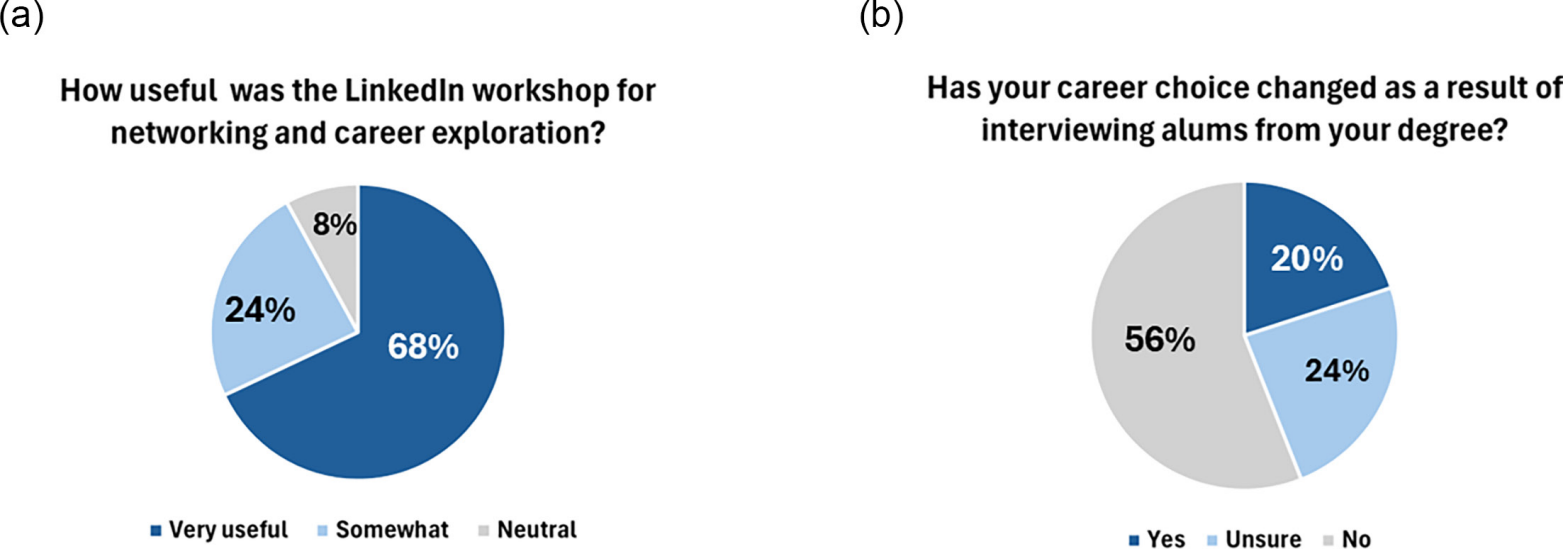
Students’ responses on the usefulness and impact on careers of the LinkedIn exercise. Percentages of respondents on questions about how useful the workshop was and what influence the interviews from session 2 had on the student career direction, taken from the post-workshop online questionnaire.

Building on activities from the teaching sessions, students were asked to reflect on whether their usage of LinkedIn would change because of the workshop, and reassuringly, 88% of respondents did confirm they would use the platform more (Fig. 4c). Finally, as the aim of this exercise was to understand whether students found this workshop useful in supporting their career explorations and networking and whether it had influenced a change in career pathway upon graduation, this result is of particular interest (Fig. 5).

Encouragingly, 92% of students reported this workshop to be very useful or somewhat useful in helping them explore their employment options and to help them network with professionals in their field (Fig. 5a). Moreover, this workshop also influenced 20% of this cohort to reconsider their career choice following the interview with their alum (Fig. 5b), demonstrating that the workshop was successful in introducing the networking platform as a tool students can utilize for networking with professionals in their field and supporting their understanding of the career opportunities open to them following graduation.

## Discussion

Students in higher education have more access to digital technologies through the online environment than ever before, providing increased opportunities for their employability but also presenting more areas in which they need development. Despite engagement with this environment from an earlier age, students may not inherently have knowledge about effective and professional use of that environment, leading to low initial levels of confidence in their abilities, as reported here and in previous studies [13]. From an educator’s perspective, this disparity presents an opportunity to both support students’ initial career development during university, while also providing real-world tools needed for the workplace [14]. However, straightforward introductions and supported engagement with the platform had clear increases in confidence and, hopefully, continued engagement going forward. As the students in this study were in the third year of their 4-year degree, embedding a related session in their final-year coursework would have considerable gains in supporting long-term use post-university. Given the digital and transferable skills developed during sessions such as these, another way to support students’ long-term use is to link with skills-tracking frameworks or portfolio tools that help students record their academic development over multiple years during their degrees [15].

While social media platforms like Snapchat and TikTok are extremely popular amongst young people [16], their primary use is social rather than professional, and although social media experience with these platforms can be beneficial as skills within professional organizations (i.e. positions such as communications officers), these platforms are not specifically designed to support career development and professional networking, which have been found to be extremely important within academic and post-academic situations [17]. LinkedIn was launched in 2003 and remains one of the oldest professional networking sites that has maintained popularity, likely due to its accessibility and clear focus on making and maintaining professional connections. The lack of a word count for posts, the potential to incorporate a range of media types such as videos or images and the ability to directly connect these with previous experience and positions provide an excellent set of engaging, relevant resources in one platform. The flexibility in creating closed groups alongside publicly available ones also provides options for maintaining both more directed connections and having public reach.

LinkedIn provides a platform for developing professional connections that can support students as they navigate the next steps in their career journey, helping with their transition into employment and ultimately career success [18]. In addition, because it does not require programming and offers a good level of features within the free version, it is a widely accessible and highly intuitive platform to use, expanding the reach globally and across most employment sectors. However, given the broad functionality supported, structured training is essential in helping students to understand how and why this platform should be used. Students were initially aware of the job search feature, but their understanding of many of LinkedIn’s other functions, some of which were arguably more important, was low initially, with some thinking the platform should only be used following graduation [19]. Therefore, supporting student engagement with this professional platform through staff involvement, through a combination of instruction and applied exercises, highlights how LinkedIn can be utilized for much more, including the creation of a personal portfolio that enables students to showcase their skills and achievements [20]. Better engagement during their degrees should also support them in using LinkedIn while in their professional careers, not just during the more initial career stages of searching and applying for positions. Embedding the ‘LinkedIn to Get In’ workshop into the Microbiology and Immunology undergraduate curriculum helped to support student development in this area by increasing student awareness of the wider benefits that LinkedIn has to offer, whilst also increasing student confidence in using the site to network with professionals in the field.

While alumni engagement and mentoring schemes are well-established practices in higher education, the ‘LinkedIn to Get In’ workshop offers a distinctive and scalable model that integrates digital networking into the curriculum in a structured and student-led way. Unlike traditional alumni talks, which are often passive and one-off events covering a range of disciplinary employers, this approach actively engages students in professional dialogue through guided interviews and reflective presentations. Although not evaluated in this study, the intangible connection between students and alums created through pursuing the same degrees fairly recently also may have made the transition from the students’ current position to that of a graduate in the workplace seem a bit more achievable, as those alums would have been in the same position as the students a few years previously. Further evaluation of this exercise through focus groups or follow-up interviews would be helpful in exploring the extent of this connection in aspects such as continued engagement and confidence in using this platform. Moreover, the use of closed LinkedIn groups creates a trusted space for interaction, while the pairing of students with alumni based on shared academic backgrounds fosters meaningful connections. Crucially, this model is low-cost, requiring minimal infrastructure, and can be rapidly implemented across a range of disciplines and institutions. The simplicity and adaptability of LinkedIn make it an attractive option for educators seeking to embed employability into their teaching in a way that is both impactful and achievable and highly relevant to graduates’ careers.

Continued engagement is likely, as indicated by students’ intentions of increasing activity on the platform by both commenting on posts and sharing their own content more. By doing this, students are raising awareness of their professional profile and demonstrating their experience and knowledge to others in the area, which can potentially lead to them making new connections and building their network [20]. However, understanding the levels of continued engagement with LinkedIn after graduation would be important in allowing us to further evaluate the longer-term impact that this workshop has on student confidence, professional networking and employability. The findings from this study align with previous work that also argues the integration of LinkedIn into the curriculum provides valuable networking opportunities and offers a platform for students to stand out from their peers in an ever-increasing competitive job market [21,22]. In addition to networking with prospective employers, LinkedIn also provides opportunities to engage with recruiters and gain access to unadvertised or informal job opportunities, offering insights into upcoming roles that may not appear on conventional job platforms. Because LinkedIn is also used by employers to verify and cross-check references for applicants, having a current and professional image on this platform is important for graduates entering the workplace [13]. Modern recruitment processes like this offer insights to students who may not be actively seeking a new role but are open to new opportunities, highlighting another benefit of embedding LinkedIn into the undergraduate curriculum to help support career awareness.

In conclusion, this study underscores the value of experiential learning and the need for practical workshops that go beyond didactic teaching when focusing on the development of digital and transferable skills. By creating a trusted and supportive environment for students to explore and utilize LinkedIn, via the closed group function, educators can facilitate meaningful learning experiences that translate into real-world skills that will support student career development over the long term and are essential for post-university success. Future pedagogical approaches should continue to incorporate such interactive and practical elements to enhance students’ professional readiness.

## Supplementary material

10.1099/acmi.0.001125.v3 Uncited Supplementary Material 1.
